# Parameters of simultaneous ^18^F-FDG-PET/MRI predict tumor stage and several histopathological features in uterine cervical cancer

**DOI:** 10.18632/oncotarget.16043

**Published:** 2017-03-09

**Authors:** Alexey Surov, Hans Jonas Meyer, Stefan Schob, Anne-Kathrin Höhn, Kristina Bremicker, Marc Exner, Patrick Stumpp, Sandra Purz

**Affiliations:** ^1^ Department of Diagnostic and Interventional Radiology, University Hospital of Leipzig, 04103 Leipzig, Germany; ^2^ Department of Pathology University Hospital of Leipzig, 04103 Leipzig, Germany; ^3^ Department of Nuclear Medicine, University Hospital of Leipzig, 04103 Leipzig, Germany

**Keywords:** uterine cervical cancer, PET, DWI, cell count, KI 67

## Abstract

The purpose of this study was to analyze associations between apparent diffusion coefficient (ADC) and standardized uptake values (SUV) values and different histopathological parameters in uterine cervical cancer. 21 patients with primary uterine cervical cancer were involved into the study. All patients underwent a whole body simultaneous^18^F-FDG PET/MRI. Mean and maximum SUV were noted (SUV_mean_ and SUV_max_). In all tumors minimal, mean, and maximal ADC values (ADC_min_, ADC_mean_, and ADC_max_) were estimated. Combined parameters were calculated: SUV_max_/SUV_mean_, ADC_min_/ ADC_mean_, SUV_max_/ADC_min_ and SUV_max_/ADC_mean_. In all cases the diagnosis was confirmed histopathologically by tumor biopsy. Histological slices were stained by hematoxilin and eosin, MIB 1 monoclonal antibody, and p16. All histopathological images were digitalized and analyzed by using a ImageJ software 1.48v. The following parameters were estimated: cell count, proliferation index KI 67, total and average nucleic areas, epithelial and stromal areas. Spearman's correlation coefficient was used to analyze associations between ADC and SUV values and histological parameters. *P value*s ≤ 0.05 were considered as statistically significant. ADC_min_ and ADC_min_/ ADC_mean_ were statistically significant lower in N positive tumors. KI 67 correlated statistically significant with SUV_max_ (r = 0.59, *p* = 0.005), SUV_mean_ (0.45, *p* = 0.04), ADC_min_ (r = −0.48, *p* = 0.03), SUV_max_/ADC_min_ (r = 0.71, *p* = 0.001), SUV_max_/ADC_mean_ (0.75, *p* = 0.001). SUV_max_ correlated well with epithelial area (r = 0.71, *p* = 0.001) and stromal areas (r = −0.71, *p* = 0.001). SUV values, ADC_min_, SUV_max_/ADC_min_ and SUV_max_/ADC_mean_ correlated statistically significant with KI 67 and can be used to estimate the proliferation potential of tumors. SUV values correlated strong with epithelial area of tumor reflected metabolic active areas.

## INTRODUCTION

Integration of magnetic resonance imaging (MRI) and ^18^F-fluorodesoxyglucose positron emission tomography (^18^F-FDG-PET) into one diagnostic system has been reported as high beneficial for investigation of different pelvic malignancies [1, 2]. It has been shown that PET/MRI demonstrated a high resolution morphological information based on MRI combined with metabolic data derived from the PET component [1, 3–6]. Furthermore, Queiroz *et al*. reported that PET/MRI accuracy was statistically superior to PET/CT for primary tumor delineation, especially in cases with cervical and endometrial cancer [2]. In addition, the authors postulated that PET/MRI may be the preferred imaging modality for staging cervical and endometrial tumors [2].

MRI can be completed by diffusion weighted imaging (DWI), which provides additional information regarding tumor texture, such as cellularity and proliferation potential [7, 8]. According to the literature, combination of apparent diffusion coefficient (ADC) as product of DWI and standardized uptake value (SUV) has been established as a useful tool in detection and staging of different pelvic tumors [9–11]. For example, previous reports suggested that DWI and SUV can predict T-and N-stage of cervical cancer [3]. Furthermore, both parameters have been reported to be useful for treatment monitoring, as well as the prediction of clinical outcome [7]. Finally, some authors observed significant correlations between SUV and ADC values in uterine cervical cancer and postulated that DWI and ^18^F-FDG-PET might play a complementary role for the clinical assessment of this malignancy [8, 11].

These findings may base on possible associations between PET, DWI and histopathological parameters in several malignancies. Some previous studies indicated that ADC and SUV reflect different aspects of tumor biology [8, 12]. For example, in head and neck cancer it has been shown that SUV and ADC correlated with different histopathological findings and, therefore, can be used as complementary biological markers [12].

We hypothesized that in uterine cervical cancer PET and DWI are also associated with histopathology and can predict biological features of tumors and tumor behavior. This is very important because to the fact that uterine cervical cancer is one of the most frequent malignancies diagnosed in women with high recurrence and 5-year mortality rates. To the best of our knowledge, no previous studies investigated this question. Therefore, the purpose of this study was to analyze possible associations between ADC and SUV values and different histopathological parameters in uterine cervical cancer.

## RESULTS

The clinical characteristics of the involved patients are shown in Table 1. In most cases (*n* = 18, 85.7%) squamous cell carcinoma was diagnosed. The grade of cell differentiation was well (G1) in one patient (4.8%), moderate (G2) in 12 cases (57.1%), and poor (G3) in 8 (38.1%). Most frequently T stages 2b and 4a were diagnosed (Table 1). Seven patients (33.3%) were staged as N0, 13 (61.9%) as N1, and one (4.8%) as N2. Furthermore, distant metastases were found in 8 (38.1%) patients (Table 1).

**Table 1:** Clinical data of the investigated patients

Case	Age	T stage	N Stage	M Stage
1	63	2b	1	0
2	76	4a	0	0
3	65	2b	0	0
4	63	4a	1	1
5	34	2b	1	0
6	57	4a	1	1
7	77	2b	1	1
8	50	1b	0	0
9	53	2b	0	0
10	32	4a	1	0
11	32	2b	0	0
12	54	3a	2	0
13	57	2a	1	1
14	79	4b	1	0
15	52	4b	0	0
16	37	2b	1	1
17	72	4a	0	0
18	46	2b	1	1
19	71	4	1	1
20	50	2b	1	1
21	61	4a	1	0

The mean, median, and standard deviation values for all analyzed DWI and PET parameters are summarized in Table 2. All of them showed wide variations. There were no significant correlations between different DWI and PET parameters (Table 3).

**Table 2:** DWI and PET parameters of cervical cancer

Parameters	M ± SD	Median	Range
**SUV_max_**	21.57 ± 10.84	17.8	9.24 – 56.20
**SUV_max_**	11.60 ± 6.47	9.73	1.7 – 32
**rSUV_max_**	2.08 ± 0.95	1.8	1.51 – 5.44
**ADC_mean_, × 10^−3^ mm^2^s^−1^**	0.86 ± 0.13	0.82	0.64 – 1.18
**ADC_min_, ×10^−3^ mm^2^s^−1^**	0.58 ± 0.16	0.56	0.37 – 0.95
**ADC_max_, ×10^−3^ mm^2^s^−1^**	1.24 ± 0.25	1.20	0.77 – 2.04
**rADC_min_**	0.67 ± 0.12	0.68	0.5 – 0.85
**SUV_max_/ ADC_min_**	40.75 ± 24.85	33.58	9.73 – 112.40
**SUV_max_/ ADC_mean_**	25.42 ± 11.91	22.25	7.83 – 62.44

**Table 3:** Correlations between DWI and SUV parameters

Parameters	SUV_max_	SUV_mean_	rSUV_max_
**ADC_mean_**	*p* = 0.13	*p* = 0.02	*p* = 0.26
	*P* = 0.58	*P* = 0.94	*P* = 0.26
**ADC_min_**	*p* = −0.13	*p* = −0.19	*p* = 0.002
	*P* = 0.59	*P* = 0.42	*P* = 0.99
**ADC_max_**	*p* = 0.09	*p* = 0.12	*p* = 0.23
	*P* = 0.71	*P* = 0.62	*P* = 0.33
**rADC_min_**	*p* = −0.14	*p* = −0.19	*p* = −0.12
	*P* = 0.54	*P* = 0.39	*P* = 0.62

**Figure 1:**
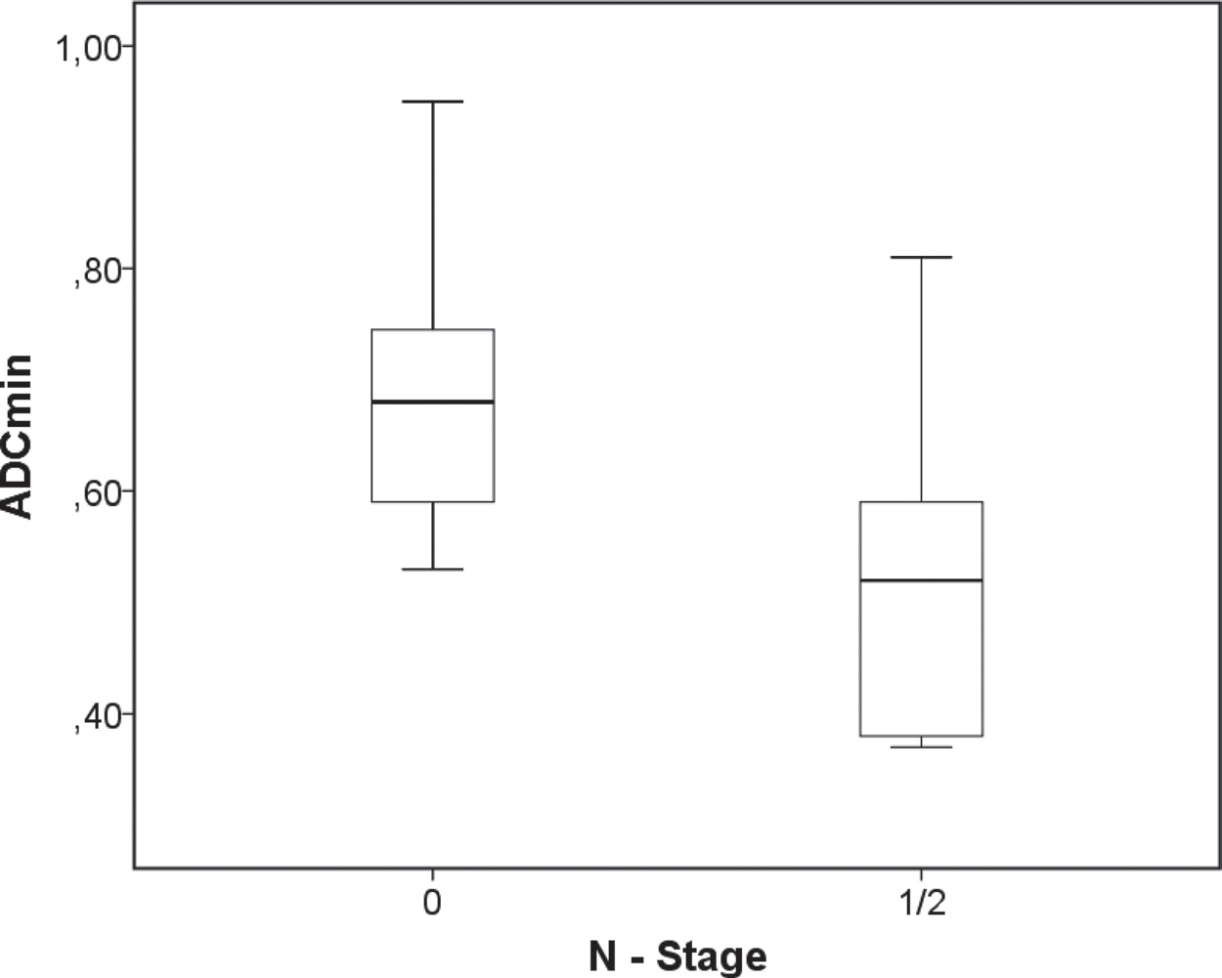
Associations between DWI and tumor stage Comparison of ADC_min_ values between N negative and N positive tumors (*p* = 0.017).

A comparison analysis of the identified PET and DWI parameters between the tumor grades and stages identified the following results. There were no significant differences in SUV and DWI values between different tumor grades (Table 4). Also the PET and DWI parameters did not differ significantly between T2 and T4 tumor stages (Table 5A). ADC_min_ and rADC_min_ were statistically significant lower in N positive tumors (*p* = 0.017 and 0.03, respectively) (Table 5B, Figure 1). Furthermore, ADC_min_ tended to be lower in M positive tumors (*p* = 0.08) (Table 5C). Additionally, the combined parameter SUV_max_/ADC_min_ had a tendency to be higher in distant metastasized cancers (*p* = 0.09).

**Table 4:** Comparison of PET and DWI values between different tumor grades

Parameters	G2 Mean ± SD	G3 Mean ± SD	ANOVA *p* values
**SUV_max_**	20.26 ± 12.15	20.86 ± 5.89	0.13
**SUV_mean_**	11.20 ± 7.37	10.83 ± 4.05	0.23
**rSUV_max_**	2.08 ± 1.06	2.11 ± 0.90	0,98
**ADC_min_**	0.56 ± 0.17	0.62 ± 0.15	0.69
**ADC_mean_**	0.85 ± 0.13	0.86 ± 0.14	0.74
**ADC_max_**	1.20.25 ± 1.25	1.26 ± 0.37	0.34
**rADC_min_/ ADC_mean_**	0.65 ± 0.12	0.72 ± 0.13	0,34
**SUV_max_/ADC_min_**	39.30 ± 25.18	38.06 ± 23.26	0.29
**SUV_max_/ADC_mean_**	24.10 ± 13.33	24.97 ± 8.31	0.26

**Table 5A:** Comparison of PET and DWI values between different tumor T stages

Parameters	T2Mean ± SD	T4Mean ± SD	ANOVA *p* values
**SUV_max_**	20.02 ± 6.64	25.34 ± 14.21	0.30
**SUV_mean_**	11.33 ± 3.82	13.28 ± 8.48	0.52
**rSUV_max_**	1.78 ± 0.14	2.09 ± 0.84	0.400
**ADC_min_**	0.59 ± 0.17	0.55 ± 0.08	0.61
**ADC_mean_**	0.85 ± 0.11	0.85 ± 0.12	0.99
**ADC_max_**	1.47 ± 0.91	1.2 ± 0.23	0.40
**rADC_min_**	0.68 ± 0.15	0.66 ± 0.08	0.55
**SUV_max_/ADC_min_**	37.92 ± 21.33	47.31 ± 29.75	0.43
**SUV_max_/ADCm_ean_**	23.87 ± 8.21	29.36 ± 14.63	0.30

**Table 5B:** Comparison of PET and DWI values between different tumor N stages

Parameters	N0	N1/2	*p* values
**SUV_max_**	20.72 ± 11.37	22.0 ± 10.98	0.81
**SUV_mean_**	10.90 ± 6.69	11.95 ± 6.58	0.73
**rSUV_max_**	2.33 ± 1.37	1.96 ± 0.69	0.39
**ADC_min_**	0.69 ± 0.15	0.52 ± 0.13	0.017
**ADC_mean_**	0.92 ± 0.17	0.83 ± 0.09	0.13
**ADC_max_**	1.23 ± 0.28	1.39 ± 0.77	0.62
**rADC_min_**	0.76 ± 0.10	0.63 ± 0.12	0.03
**SUV_max_/ADC_min_**	33.13 ± 23.23	44.56 ± 25.58	0.33
**SUV_max_/ADC_mean_**	23.39 ± 12.53	26.43 ± 11.93	0.59

**Table 5C:** Comparison of PET and DWI values between different tumor M stages

Parameters	M0	M1	*p* values
**SUV_max_**	19.53 ± 8.25	24.89 ± 14.10	0.28
**SUV_mean_**	10.06 ± 5.07	14.10 ± 8.00	0.17
**rSUV_max_**	2.28 ± 1.18	1.76 ± 0.13	0.45
**ADC_min_**	0.63 ± 0.13	0.50 ± 0.13	0.08
**ADC_mean_**	0.89 ± 0.14	0.81 ± 0.09	0.20
**ADC_max_**	1.43 ± 0.81	1.18 ± 0.12	0.41
**rADC_min_**	0.70 ± 0.11	0.64 ± 0.14	0.34
**SUV_max_/ADC_min_**	33.64 ± 16.87	52.30 ± 32.10	0.09
**SUV_max_/ADC_mean_**	22.50 ± 9.02	30.15 ± 14.98	0.16

The results of histopathological analysis are shown in Table 6. Histopathological findings varied with a wide spectrum in the patients.

**Table 6:** Estimated histopathological parameters of cervical cancer

Parameters	M ± SD	Median	Range
**Cell count**	1780 ± 334	1795	1290–2515
**Ki 67, %**	48.38 ± 18.42	49	22–76
**Total nucleic area, μm^2^**	113665 ± 32709	109711	58914–181174
**Average nucleic area, μm^2^**	63.72 ± 13.66	59.87	38.21–94.43
**Epithel area (%)**	30.57 ± 16.55	30	2–60
**Stroma area (%)**	69.43 ± 16.55	70	40–98

**Figure 2:**
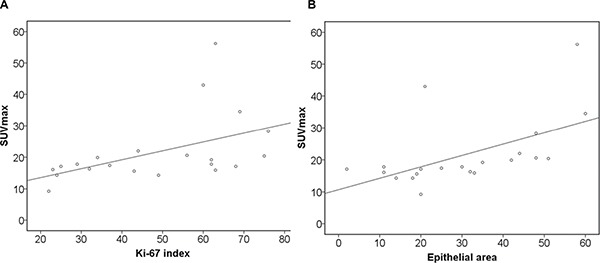
Associations between glucose metabolism and histopathological parameters (**A**) Correlation between SUV_max_ and KI 67 (*r* = 0.59, *p* = 0.005). (**B**) Correlation between SUV_max_ and epithelial area (*r* = 0.71, *p* = 0.001).

**Figure 3:**
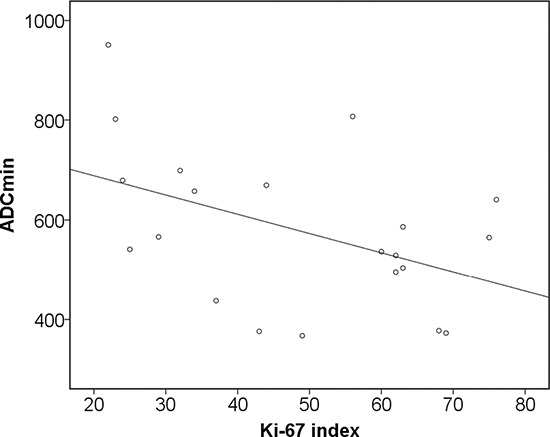
Associations between DWI and histopathology Correlation between ADC_min_ and KI 67 (*r* = −0.48, *p* = 0.03).

Furthermore, histopathological findings were correlated with PET and DWI parameters (Table 7A–7C). KI 67 correlated statistically significant with SUV_max_ (r = 0.59, *p* = 0.005), and SUV_mean_ (0.45, *p* = 0.04) (Figure 2A). SUV_max_ showed strong correlations with epithelial area (r = 0.71, *p* = 0.001) and stromal areas (r = −0.71, *p* = 0.001) (Figure 2B). Also SUV_mean_ correlated with epithelial area (r = 0.45, *p* = 0.04) and stromal areas (r = −0.45, *p* = 0.04) (Table 7A). In addition, ADC_min_ correlated inversely with KI 67 (r = −0.48, *p* = 0.03) (Table 7B, Figure 3).

**Table 7A:** Correlations between PET and histopathological parameters

Parameters	Cell count	Ki 67	Total nucleic area	Average nucleic area	Epithelial area	Stromal area
**SUV_max_**	*p* = 0.24	***p* = 0.59**	*p* = 0.29	*p* = 0.16	***p* = 0.71**	***p* = −0.71**
	*P* = 0.29	***p* = 0.005**	*P* = 0.19	*P* = 0.48	***p* = 0.001**	***p* = 0.001**
**SUV_mean_**	*p* = 0.03	***p* = 0.45**	*p* = 0.13	*p* = 0.17	***p* = 0.45**	***p* = −0.45**
	*P* = 0.88	***P* = 0.04**	*P* = 0.58	*P* = 0.45	***P* = 0.04**	***P* = 0.04**
**rSUV_max_**	*p* = 0.11	*p* = −0.09	*p* = −0.05	*p* = −0.24	*p* = 0.003	*p* = −0.003
	*P* = 0.65	*P* = 0.69	*P* = 0.83	*P* = 0.29	*P* = 0.99	*P* = 0.99

**Table 7B:** Correlations between DWI and histopathological parameters

Parameters	Cell count	Ki 67	Total nucleic area	Average nucleic area	Epithelial area	Stromal area
**ADC_mean_**	*p* = 0.01	*p* = −0.34	*p* = −0.19	*p* = −0.29	*p* = 0.31	*p* = 0.31
	*P* = 0.96	*P* = 0.14	*P* = 0.39	*P* = 0.21	*P* = 0.17	*P* = 0.17
**ADC_min_**	*p* = −0.07	***p* = −0.48**	*p* = −0.11	*p* = −0.07	*p* = 0.07	*p* = 0.07
	*P* = 0.77	***p* = 0.03**	*P* = 0.65	*P* = 0.76	*P* = 0.77	*P* = 0.77
**ADC_max_**	*p* = 0.09	*p* = −0.24	*p* = −0–16	*p* = −0.30	*p* = 0.08	*p* = −0.08
	*P* = 0.69	*P* = 0.29	*P* = 0.50	*P* = 0.18	*P* = 0.72	*P* = 0.72
**rADC_min_**	*p* = −0.17	*p* = −0.35	*p* = 0.03	*p* = 0.22	*p* = −0,88	*p* = 0,09
	*P* = 0.46	*P* = 0.12	*P* = 0.88	*P* = 0.34	*P* = 0,70	*P* = 0,70

**Table 7C:** Correlations between combined PET/DWI and histopathological parameters

Parameters	Cell count	Ki 67	Total nucleic area	Average nucleic area	Epithelial area	Stromal area
**SUV_max_/ADC_min_**	*p* = 0.149	***p* = 0.71**	*p* = 0.27	*p* = 0.19	*p* = 0.27	*p* = 0.27
	*P* = 0.52	***p* ≤ 0.001**	*P* = 0.23	*P* = 0.39	*P* = 0.24	*P* = 0.24
**SUV_max_/ADC_mean_**	*p* = 0.20	***p* = 0.75**	*p* = 0.41	*p* = 0.35	***p* = 0.49**	***p* = −0.49**
	*P* = 0.39	***p* ≤ 0.001**	*P* = 0.07	*P* = 0.12	***p* = 0.03**	***p* = 0.03**

**Figure 4:**
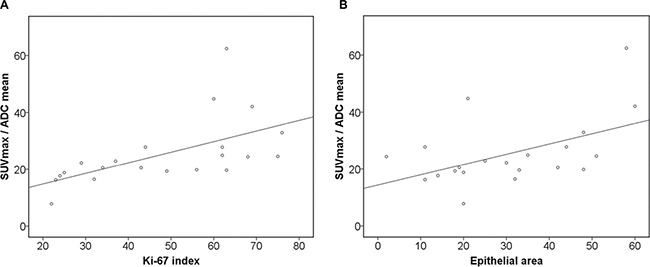
Associations between combined PET/DWI parameters and histopathology (**A**) Correlation between SUV_max_/ADC_mean_ and KI 67 (*r* = 0.75, *p* = 0.001). (**B**) Correlation between SUV_max_/ADC_mean_ and epithelial area (*r* = 0.49, *p* = 0.03)

Significant correlations were also observed between KI 67 and the calculated combined parameters SUV_max_/ADC_min_ (r = 0.71, *p* = 0.001) and SUV_max_/ADC_mean_ (0.75, *p* = 0.001) (Figure 4A and 4B). SUV_max_/ADC_mean_ showed moderate correlations with epithelial (r = 0.49, *p* = 0.03) and stromal areas (r = −0.49, *p* = 0.03). Finally, SUV_max_/ADC_mean_ tended to correlate with total nucleic area (r = 0.41, *p* = 0.07) (Table 7C).

## DISCUSSION

The present study identified significant associations between tumor stage, histopathological findings and parameters from simultaneous PET/MRI in uterine cervical cancer.

According to the literature, PET and DWI are independent imaging modalities, which reflect different clinical and histological features in several tumors [8, 12]. For example, Preda *et al*. reported that both SUV and ADC values can be used as prognosis factors in patients with head and neck cancer [9]. Other authors confirmed these results [10].

In contrast to other malignancies, there were only few studies regarding PET and DWI findings in uterine cervical cancer. Previously, some authors identified significant associations between PET and DWI parameters in uterine cervical cancer [3, 8]. For instance, Brandmaier *et al*. found significant inverse correlations between SUV_max_ and ADC_min_ (r = −0.532, *p* = 0.05) and between SUV_mean_ and ADC_min_ (r = −0.403, *p* = 0.03). in primary tumors [8]. Furthermore, the identified correlations were stronger in recurrent tumors: SUV_max_ and ADC_min_ (r = −0.747, *p* = 0.002) and SUV_mean_ and ADC_min_ (r = −0.773, *p* = 0.001) [8]. Also Grueneisen *et al*. studied correlations between SUV_max_ and ADC_min_ in primary and recurrent cervical cancer [3]. It has been shown, however, that the parameters correlated well in primary tumors and associated primary lymph node metastases, but not in recurrent cancer lesions [3]. In contrast to these reports, in the study of Ho *et al*. no significant correlations between ADC_min_ as well ADC_mean_ and SUV_max_ or SUV_mean_ were identified [11]. Nevertheless, the authors calculated two new indexes, namely rADC_min_ as a ratio ADC_min_/ADC_mean_ and rSUV_max_ as a ratio SUV_max_/SUV_mean_ [11]. It has been shown that both parameters correlated well together in adenocarcinomas and adenosquamous tumors but not in squamous carcinomas [11].

In our study, no significant correlations between PET and ADC parameters were found. In addition, also the calculated indexes rADC_min_ and and rSUV_max_ did not correlate together. This finding is in agreement with the results of Ho *et al*. [11] because of the fact that our patients had predominantly squamous cell carcinomas.

Some previous reports indicated that PET and DWI parameters can be used as predictor of tumor stage and grading. So Micco *et al*. observed significant correlations between ADC_mean_, SUV_max_, metabolic tumor volume, total lesion glycolysis and occurrence of lymph node metastasis [10]. Grueneisen *et al*. found that ADC_min_ was statistically significant lower in 2b-4 T stage tumors in comparison to T1-2a carcinomas [3]. Furthermore, ADC_min_ had a tendency to be lower in tumors with nodal metastases [3]. Other authors confirmed these findings [10, 13].

In the study of Husby *et al*, ADC was associated with deep myometrial invasion in cervical cancer [14]. It has been shown that invasive tumors had significantly lower mean tumor ADC values in comparison to tumors without myometrial invasion [14]. Similar results were reported for cervical cancer with parametral invasion [15].

Moreover, according to the literature, ADC can also distinguish different histological tumor types of cervical cancer [16]. For example, Liu *et al*. reported that mean ADC value and minimum ADC value of squamous cell carcinoma were significantly lower than that of adenocarcinoma [16]. However, Grueneisen did not found significant differences in SUV as well ADC values between squamous cell carcinomas and adenocarcinomas [3].

Previous reports indicated that SUV and ADC can be used to distinguish well or moderately differentiated carcinomas and poorly differentiated cervical tumors [3].

For example, it has been shown that G3 tumors had statistically significant lower ADC_min_ values and higher SUV_max_ and SUV_mean_ values [3]. Micco *et al*, however, could not identify significant differences in SUV and ADC values between several tumor grades [10].

In the present study, also an analysis of PET and DWI parameters in dependency on tumor stage and grading was performed. No significant associations between different DWI and PET parameters as well calculated indexes in moderately and poorly differentiated tumors were identified. This finding indicates that tumor grading does not influence PET and DWI in uterine cervical cancer. However, we found significant associations between PET and DWI parameters and different tumor stages. Firstly, ADC_min_ and rADC_min_ were statistically significant lower in tumors with nodal metastases. Secondly, ADC_min_ tended to be lower (*p* = 0.082) in tumors with distant metastases (M stage). Interestingly, also SUV_max_/ADC_min_ had a tendency to be higher (*p* = 0.095) in M positive carcinomas. To the best of our knowledge, associations between imaging and M stage have not been reported previously in uterine cervical cancer. These findings have a high clinical relevance and suggests that ADC_min_ as well SUV_max_/ADC_min_ may be used as M-stage markers. No significant differences were identified between the tumors in dependency to T stage. It may be explained to the fact that, in contrast to previous reports, our patients had predominantly 2b and 4 stages, i.e. advanced tumors. Overall, our results confirmed the hypothesis of some previous studies that PET and DWI parameters can be used as additional predictors for tumor stage.

Presumably, the identified associations base on associations between PET and DWI parameters and histopathological features. In contrast to other tumors, such as head and neck cancers [12], breast carcinoma [17] or lung cancer [18] there were no reports regarding possible correlations between PET/MRI and histopathological findings in uterine cervical cancer.

In our study, a complex analysis of relationships between PET, DWI, and histopathology was performed. Thereby several significant correlations between the investigated parameters were identified. Firstly, ADC_min_ and SUV_max_ as well SUV_mean_ correlated significantly with KI 67. Therefore, these parameters can be used to assess proliferation potential in cervical cancer. Secondly, PET parameters did not reflect cell count of the investigated tumors. However, the present study demonstrated well correlations between PET parameters and tumor architecture, in particular epithelial and stromal areas. This finding is very interestingly and may explain missing correlations between SUV fractions and cellularity in uterine cervical cancer and similar results of a previous analysis regarding squamous cell carcinomas in the head and neck region [12]. In fact, each tumor consists on tumor cells and stroma. Only tumor cells have high metabolic activity and influence PET parameters. Therefore, SUV_max_ and SUV_mean_ reflect tumor cell count/area, but not overall cell count/area.

Thirdly, in our study, no significant correlations between different ADC values and cell count were detected. This finding is difficult to explain. According to the literature, in most reported malignancies, different ADC values correlated significantly with cell count [19–22]. It has been shown that especially ADC_min_ reflected tumor cellularity [19, 20, 23]. However, there were several lesions, in which also no significant correlations between DWI parameters and cell count were found [29]. For example, Wu *et al*. did not find any correlations between ADC values and tissue cellularity in different lymphomas [24]. It may be related to the fact that not only cellularity but other histopathological features such as architectural structure [25], extracellulary matrix [20] or nucleic areas [18, 19, 25] may play a role here.

Our study showed that the calculated combined parameters SUV_max_/ADC_mean_ and SUV_max_/ADC_min_ demonstrated significant associations with KI 67, epithelial and stromal areas. Moreover, the correlations KI 67 vs the combined parameters were stronger than those vs ADC_min_, SUV_max_ or SUV_mean_. Therefore, these combined parameters can better predict proliferation potential of uterine cervical cancer.

Furthermore, the ratio SUV_max_/ADC_mean_ tended to correlate with total nucleic area. Nuclear size was reported to be a prognostic indicator in several malignancies [26]. It has also been shown that lesions with large nucleic areas had a worse prognosis [26]. The phenomena identified in our study confirmed the assumption that PET and DWI parameters complement one another and they can be combined together.

The present study is limited to the relatively small number of patients. Clearly, further investigations with more patients are needed to confirm the identified associations between clinical, imaging and histopathological parameters.

In conclusion, our results quantitatively demonstrated significant correlations between PET and DWI parameters and different histopathological features in uterine cervical cancer. N positive tumors showed statistically significant lower ADC_min_ and rADC_min_ values. Both SUV values, ADC_min_, as well combined parameters SUV_max_/ADC_min_ and SUV_max_/ADC_mean_ correlated statistically significant with KI 67 and can be used to estimate the proliferation potential of tumors. Finally, both SUV values correlated strong with epithelial area of tumor and, therefore, reflected metabolic active areas but not overall tumor cell count.

## MATERIALS AND METHODS

This prospective study was approved by the institutional review board (Ethic Committee of the Medical Faculty, University of Leipzig) and all patients gave written informed consent.

### Patients

Overall, 21 patients (mean age, 56.2 ± 14.5 years; median age, 57 years; range, 32-79 years) with histologically proven primary uterine cervical cancer were involved into the study (Table 1).

### Whole-body PET/MRI

All 21 patients underwent a whole body simultaneous^18^F-FDG PET/MRI (Magnetom Biograph mMR - Biograph, Siemens Health Care Sector, Erlangen, Germany) which was performed from the upper thigh to the skull with 5 minutes per bed position. PET images were reconstructed using the iterative ordered subset expectation maximization algorithm with 3 iterations and 21 subsets, a Gaussian filter with 4 mm full width at half maximum (FWHM), and a 256 × 256 image matrix. Attenuation correction of the PET data was performed using a four-tissue (fat, soft tissue, air, background) model attenuation map, which was generated from a Dixon-Vibe MR sequence as previously described by Martinez-Möller et al. 2009 [27].

Radiotracer administration was performed intravenously after a fasting period of at least 6 hours with a body weight-adapted dose of ^18^F-FDG (4 MBq/kg, range: 152 – 442 MBq, mean±std: 285±70 MBq). PET/MRI image acquisition started on average 122 minutes after ^18^F-FDG application. Due to radiotracer elimination via the urinary tract, which may influence evaluation of pelvic PET images, all patients received a bladder catheter prior to PET/MRI examination.

### Pelvic MRI

Additionally, pelvic MRI was obtained in all cases. For pelvic MRI the following sequences were applied: a transverse T2 turbo spin echo (TSE) sequence, a sagittal T2 TSE sequence, a transverse T1 TSE sequence, a transverse fat saturated T1 TSE after intravenous application of contrast medium (0.1 mmol/kg body weight Gadobutrol, Bayer Healthcare, Germany), a sagittal post contrast T1 TSE, and a transverse diffusion-weighted echo-planar imaging (EPI) sequence by using two b-values: b0 and b1000 s/mm^2^.

Table 8 provides detailed information for all sequences of the pelvic study protocol.

**Table 8:** Sequences used in the study

Sequences	TR, ms	TE, ms	Flip angle	Slice thickness
Transverse T2 TSE	5590	105	140	7
Sagitta l T2 TSE	4110	131	150	3
Transverse T1 TSE	1310	12	140	7
Transverse post contrast fat saturated T1 TSE	912	12	140	7
sagittal post contrast T1 TSE	593	12	140	5
EPI 2d DWI	4900	105	90	3

### Image analysis

Imaging data were analyzed with dedicated viewing software (syngo.via, Siemens Health Care, Erlangen Germany). This was performed by two board certified physicians, a radiologist with 7 years’ experience in gynecological imaging and a nuclear medicine specialist with 8 years’ experience in oncological hybrid imaging.

**Figure 5:**
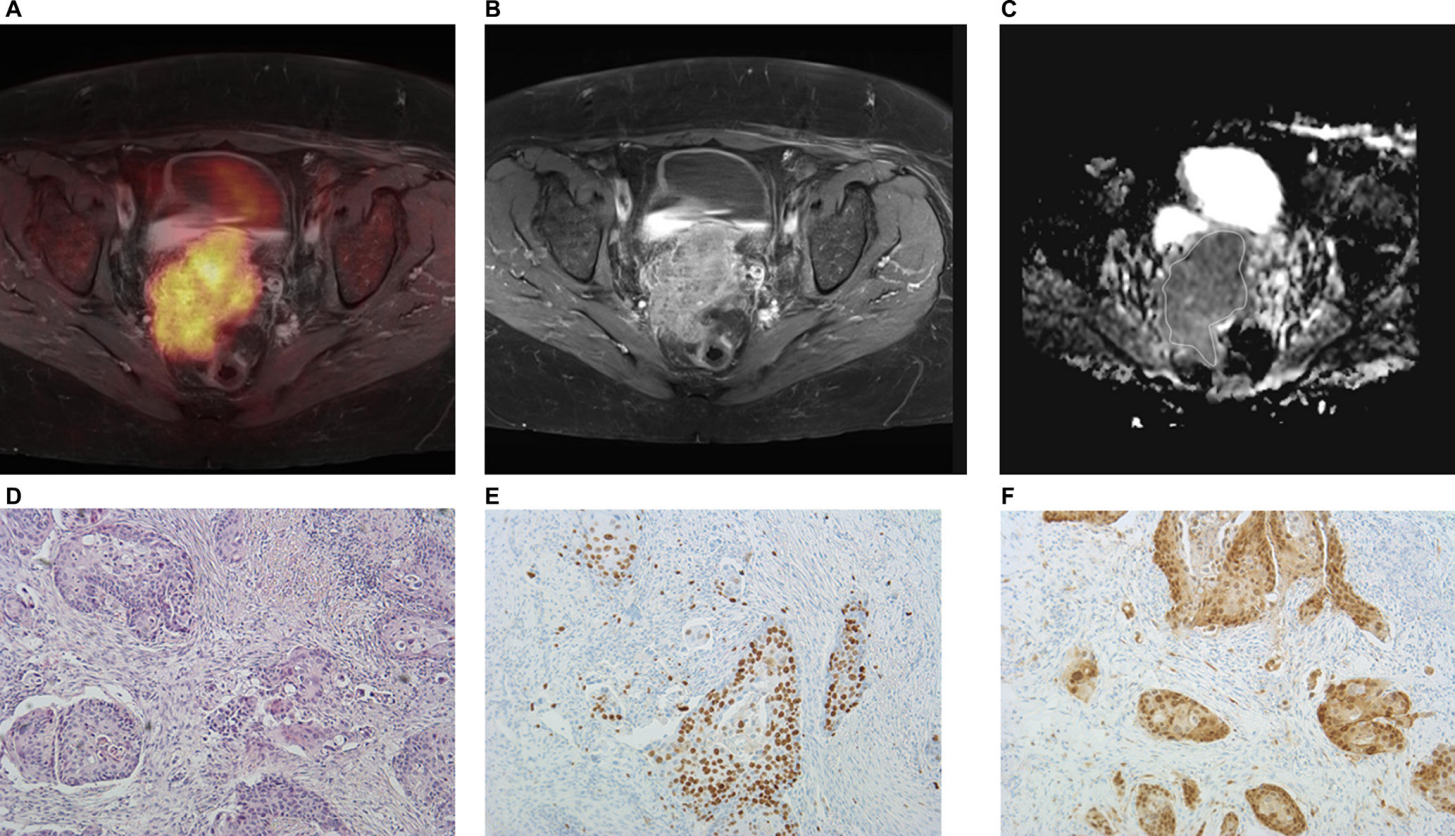
Imaging and histopathological findings in a patient with T4a N0 M0 uterine cervical cancer (**A**) fused ^18^F-FDG-PET/T1 weighted TSE MR image of the lesion, SUV_max_ = 16.3, SUV_mean_ = 8.68. (**B**) Postcontrast T1 weighted TSE showing a large lesion in the uterine cervix. (**C**) ADC map of the tumor. The ADC values (× 10^−3^ mm^2^s^−1^) of the lesion are as follows: ADC_min_ = 0.70, ADC_mean_ = 0.99, and ADC_max_ = 1.33. (**D**–**F**). Histopathological images. H&E image (D): cell count is 1971, total nucleic area = 108240 μm^2^, average nucleic area = 54.97 μm^2^. (E) Immunohistochemical stain (MIB-1 monoclonal antibody). Ki 67 index is 32%. (F) Immunohistochemical stain (p16 antibody). Epithelial area is 32% and stromal area is 68%.

For PET-imaging, focal lesions with glucose uptake greater than the surrounding tissue were considered suspicious for malignancy. A volume of interest (VOI) was drawn around these lesions using the VOI-isocontour function of the software with a threshold of 40% of SUV_max_ (Figure 5A and 5B). Mean and maximum standardized uptake values were noted (SUV_mean_ and SUV_max_). Furthermore, in every case a relative SUV_max_ as a ratio SUV_max_/SUV_mean_ was calculated as reported previously [11, 28].

Only pelvic MR investigations were analyzed in the study. Detected primary cervical cancers were analyzed with cognitive fusion of T2 weighted images and DWI images. ADC maps were automatically generated by the scanner software. For calculation of diffusion parameters of the tumor, the borders of tumor tissue were marked freehand with a polygonal region of interest (ROI) in each slice on the ADC maps (Figure 5C). In all tumors minimal ADC values (ADC_min_), mean ADC values (ADC_mean_), and maximal ADC values (ADC_max_) were estimated. Additionally, a relative ADC_min_ as a ratio ADC_min_/ ADC_mean_ was calculated [11].

Furthermore, in each case combined parameters PET and DWI were calculated as follows: SUV_max_ divided by ADC_min_ (SUV_max_/ADC_min_) and SUV_max_ divided by ADC_mean_ (SUV_max_/ADC_mean_) [12, 29].

### Histopathological analysis

All histopathological images were analyzed by one pathologist (10 years of experience).

In all cases the diagnosis was confirmed histopathologically by tumor biopsy. The biopsy specimens were deparaffinized, rehydrated and cut into 5 μm slices. Furthermore, the histological slices were stained by hematoxilin and eosin (H&E), MIB 1 monoclonal antibody (DakoCytomation, Denmark), and p16 (Cintec Histology, Roche, Germany) according to previous descriptions [30].

All histopathological images were digitalized with a research microscope Jenalumar and camera Diagnostic instruments 4.2 (Zeiss, Jena, Germany) and saved as uncompressed Tagged Image File Format (TIFF). The digitalized images were analyzed by using ImageJ software 1.48v (National Institutes of Health Image program) with a Windows operating system [31]. All images were converted to a black and white binary image by setting the image threshold as reported previously [23, 31]. The threshold selected image parts were further analyzed using the Analyze Particles tool [23]. The following histopathological parameters were estimated: cell count, proliferation index KI 67, total nucleic area, average nucleic area, and epithelial/stromal areas of tumors. Cell count was estimated as a number of all nuclei on H&E stained images (Figure 5D). Proliferation index KI 67 (%) was calculated as percentage of stained nuclei on MIB 1 monoclonal antibody stained images (Figure 5E). Thereby the areas with the highest number of positive tumor nuclei were selected. Total nucleic area (μm^2^) was given as area of stained nuclei on H&E stained images. Furthermore, also an average nucleic area (μm^2^) as a total nucleic area divided by number of nuclei was calculated. In addition, stained area on p16-stained images divided by total area of image x 100% (in every case, the total area of image was 0.16 mm^2^) was estimated. Because to the fact that only epithelial cells can be stained by p16 [30], the stained area was acquired as epithelial area of tumor (Figure 5F). Finally, stromal area of tumor (%) as a nonstained area on p16-stained images divided by total area of image x 100% was also calculated.

In every case, 2 five high power fields (0.16 mm^2^ per field) with a magnification of x400 were analysed.

### Statistical analysis

Statistical analysis was performed using IBM SPSS 20™ (SPSS Inc., Chicago, IL, USA). Collected data were evaluated by means of descriptive statistics (absolute and relative frequencies). All measurements were non-normally distributed according to Kolmogorov-Smirnov-test. Spearman's correlation coefficient was used to analyze associations between ADC and SUV values and histological parameters. *P* values ≤ 0.05 were considered as statistically significant.
